# The reality of transdisciplinarity: a framework-based self-reflection from science and practice leaders

**DOI:** 10.1007/s11625-015-0328-2

**Published:** 2015-08-13

**Authors:** Claudia R. Binder, Iris Absenger-Helmli, Thorsten Schilling

**Affiliations:** 1 Chair for Human-Environment-Relations, Department of Geography, Ludwig-Maximilians-University Munich, Luisenstraße 37, 80333 Munich, Germany; 2 EU Leader Energieregion Weiz-Gleisdorf GmbH, Franz-Pichler-Straße 32, 8160 Weiz, Austria

**Keywords:** Transdisciplinarity, Reality check, Self-reflection, Framework, Energy regions

## Abstract

This paper provided results of a framework-based self-reflection process conducted by the science and the practice leaders of two transdisciplinary projects realized in co-leadership from 2011 until 2014. It analyzes from the perspectives of the science and practice leaders for the whole research process including preparation, research, and follow-up phase, the (1) transdisciplinarity component of each module (in %); (2) outputs generated (tangible and intangible); (3) relevance of output for science and practice (qualitative ranking); (4) impacts emerging from the outputs (tangible and intangible); and (5) outcomes emerging from the impacts (tangible and intangible). Furthermore, the research process was reflected by practice and science project leaders and critical aspects identified. We found that first, a transdisciplinary research process might contribute to regional demands if it is carried out “timely.” Timeliness includes (1) the need from the perspective of the practice partners and the scientific community, (2) the willingness of the co-leaders to develop the project together, and (3) the fundamental organizational support. This was the case in our project where the results directly impacted the further development of the project. Second, a truly lived co-leadership consisting of clearly defined and lived roles and responsibilities, common definition and alignment of the goals, and acceptance of the differences in needs by practice and science leads to a trustful cooperation. Third, a good communication structure within the teams and between the practice and science teams allows to anticipating and overcoming problems at the practice-science interface leading to mutual learning and experience building.

## Introduction

Society is facing major global challenges like the damage or loss of resources or climate change (Ostrom 2009), which might threaten the livelihood of this very society. Scholars have found that to deal with these complex challenges, inputs from different academic disciplines alone are not sufficient (Kates et al. 2001; Zscheischler et al. 2014). Moreover, they argue that beyond interdisciplinary research, trying to solve these problems by integrating knowledge across different disciplines, approaches have to be developed that support the integration of knowledge from actors who are outside of the research process itself.

Transdisciplinary research (TDR) has been claimed to be able to do so (Hirsch Hadorn et al. 2008a). This type of research (1) substantially includes actors from outside academia, (2) deals with socially relevant real-world, ‘wicked’ problems, (3) aims at mutual learning processes by including the knowledge not only from different scientific disciplines, but also from actors outside science, and (4) creates knowledge that is solution-oriented in a way that it generates results that are relevant to both practice and science (Defila et al. 2006; Scholz et al. 2006; Lang et al. 2012; Mauser et al. 2013). Importantly, the co-generation of knowledge in transdisciplinary research is not a linear process, but occurs in an iterative, reflexive cycle (Lang et al. 2012).

Investigations abound into the nature, processes, and potential (in) effectiveness of TDR. Thereby, we identified three strands of research. A first strand has dealt with the question of how to set up TD projects. Here, several ideal–typical conceptual models have been developed which prescribe how transdisciplinary projects should be structured and how the mutual learning process should be designed (Scholz et al. 2006; Scholz 2000; Lang et al. 2012; Wiek 2007; Bergmann et al. 2005; Carew and Wickson 2010). While most of these models reveal differences in their understanding of an ideal–typical TD process, there still exist some common denominators. Many authors agree that a typical TD project consists of three phases: The first phase relates to problem framing and team building. Scientists and practitioners clarify their perspectives, problems, and expectations and try to agree on a common set of goals to frame the project. In the second phase, project partners focus on project work and (co)-generation of knowledge and there can be different types of actor involvement. This said, not every aspect of the process in phase two has to be transdisciplinary. Even if the second phase consists of (inter-)disciplinary modules, each (disciplinary) part has to contribute to the commonly defined goals of the first phase. The third phase revolves around knowledge integration. This includes the process of making the results useful for both scientists and practitioners. For practitioners, the results should contribute to solving societal problems or to inducing or supporting societal transformations. Scientists, on the other hand, look for new insights regarding methodology, theory development, or empirical evidence (Lang et al. 2012; Bergmann et al. 2005; Scholz 2000; Carew and Wickson 2010; Scholz et al. 2006). To what extent these three phases have been implemented and have led to the desired effects in science and practice is highly debated (Zscheischler et al. 2014; Carew and Wickson 2010; Wolf et al. 2013).

A second strand has addressed the question of how to evaluate the success of TDR (Defila and Di Giulio 1999; Bergmann et al. 2005; Jahn and Keil 2015; Carew and Wickson 2010). For example, Walter et al. (2007) and Wolf et al. (2013) present several evaluation frameworks for assessing the social effects of TD projects and Klein (2008) identifies several criteria for the evaluation of inter- and trans-disciplinary projects. Nevertheless, there is consensus that more research is needed on how the different perspectives of practitioners and scientists should be included in the evaluation of TD projects (Zscheischler et al. 2014; Klein 2008).

A third strand deals with experiences of researchers with real-world projects and forms the foundation to overcome the above-mentioned knowledge gaps. Thereby, scholars have reflected on the TD process itself and have published accounts of their own experiences with TDR (e.g., Tötzer et al. 2011; Antrop and Rogge 2006; Serrao-Neumann et al. 2015). However, most of these self-reflective case studies lack any conceptual frameworks to structure the reflection process itself. In this paper, we argue that the use of such a framework would greatly enhance the comparability of self-reflection exercises and make the evaluation process more comprehensible and reproducible. Furthermore, self-reflection is usually carried out from a scientific perspective, which does not include the views and perspectives of local partners or co-leaders from practice (Tötzer et al. 2011; Antrop and Rogge 2006; Serrao-Neumann et al. 2015).

We provide results of a framework-based self-reflection process conducted by the science leader and the practice leader of two transdisciplinary projects realized in co-leadership from 2011 until 2014. Our perspective is thus limited to the perceptions of the two leaders of these projects. We included to where possible the views of the co-workers involved in the project. The self-reflection exercise took place a year after the projects concluded.

The two TD projects in question dealt with energy transitions in the Austrian energy region (*Energieregion*) Weiz-Gleisdorf. Covering the entire research process, including preparation, research, and follow-up, we analyze (1) the degree of transdisciplinarity of each part of the project module (in %); (2) outputs generated (tangible and intangible); (3) relevance of output for science and practice (qualitative ranking by practice and science project leaders); (4) impacts emerging from the outputs (tangible and intangible); and (5) outcomes emerging from the impacts (tangible and intangible). Finally, we discuss our results in relation to experiences made by other researchers and derive some conditions and criteria for fruitful TD projects carried out in co-leadership between science and practice.

The paper is structured as follows: “Conceptual approach and methods” section presents our conceptual approach and the methods applied for data collection and analysis. “The case study” section provides an overview of the study area and the projects themselves. “Results” section presents the results; “Discussion” section discusses the results and presents areas for further research. Finally, “Conclusion” section concludes.

## Conceptual approach and methods

### Conceptual approach

**Fig. 1:**
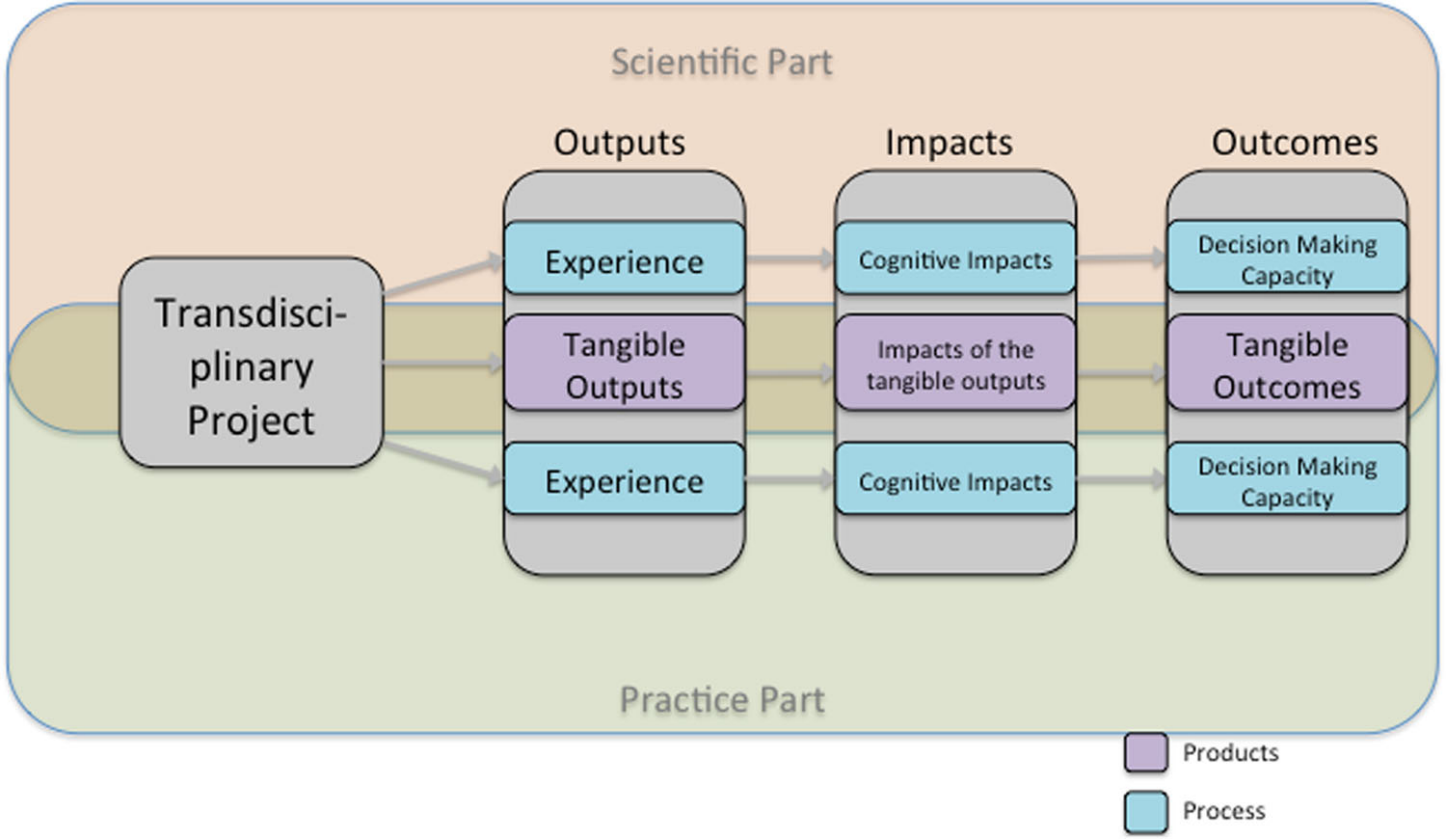
Conceptual framework used to structure the self-reflection by the science and practice leaders (adapted from Walter et al. 2007)

For the self-reflection process we adapted the framework developed by Walter et al. (2007) (Fig. 1). Each transdisciplinary project delivers outputs, impacts, and outcomes that can be further differentiated into product-related (tangible) and process-related (intangible) effects. Thereby, we define *outputs* as the immediate results of a TD project. Product-related outputs are tangible results such as reports, publications, work-shops, meetings etc. (Walter et al. 2007). Process-related outputs are intangible and largely experiential, including (1) methodological, (2) organizational, and (3) social experiences. Methodological experience captures how actors from different backgrounds become familiar with each other’s way of working, including problem definition, language, methods, and working culture (Walter et al. 2007; Beierle 1999). Organizational experience relates to the practical experience gained by planning, managing, structuring, and executing the project (Winter et al. 2006) and involves analyzing during or after the project whether or not the project plan matched the actual process. Social experience is defined as the interaction with other actors, entities, or institutions. Positive interactions build trust (as an impact of the social experience) while negative ones reduce it. That way each actor within the project tests, differentiates, and adapts their network connections over time (Walter et al. 2007; Beierle 1999; Zscheischler et al. 2014).

We define *impacts* as intermediate effects (Walter et al. 2007). Tangible impacts describe the influence that tangible outputs have on actions, decisions, plans, and measures after the project. Furthermore, the project can result in tangible impacts by producing different forms of knowledge. We distinguish three types of knowledge: (1) system knowledge, (2) target or goal knowledge, and (3) transformation knowledge (ProClim and CASS 1997). System knowledge delivers information about the structures, processes, and problems within a system. Goal or target knowledge has a more normative component. It shows the need for change and the desired goals. Transformation knowledge supports the transformation from the existing system state to a desired one. It includes knowledge about different technical, social, political, economic, cultural, and legal methods and means that facilitate the transformation process (Pohl and Hirsch Hadorn 2007). Intangible impacts are the cognitive impacts as described in Walter et al. (2007). They are the direct consequences of the different types of experiences made during the project. That is, actors might use their experiences to improve their skills such as better understanding for the viewpoint of others in future projects, more efficient project management, and develop more stable and reliable networks.

Finally, we define *outcomes* as the long-term effects, such as system changes as a consequence of a TD project (Walter et al. 2007). In contrast to Walter et al. (2007), we consider not only the fulfillment of the goals of the project, but also the implementation of the results in the study area. Furthermore, we look at the potential leverage of the projects beyond the project goals. Tangible project outcomes include the implementation of a scenario or vision, as well as political or economic consequences. Intangible outcomes relate to an increased decision-making capacity of practitioners and scientists. Since outcomes relate to long-term changes, it is often difficult to identify or predict them (Defila and Di Giulio 1999; Spaapen and van Drooge 2011). Furthermore, these changes can be consequences of multiple causes and might not be easily attributed to the TD project alone (Spaapen and van Drooge 2011).

### Methods

To facilitate the self-reflection process, we analyzed the different parts of the project by modules (work packages) as defined in the project proposal, including a preliminary phase prior to the start of the project as well as a follow-up phase. For each of these modules, we used the perspective of the practice and science leaders—who were identical for the two projects—to identify and analyze the following aspects:

- Degree of TD of each module (%)
- Outputs generated (tangible and intangible)
- Relevance of output for science and practice
- Impacts emerging from the outputs (tangible and intangible)
- Outcomes emerging from the impacts (tangible and intangible)

The first three points were assessed quantitatively, whereas the last two aspects were investigated qualitatively. The degree of TD relates to the transdisciplinary co-generation of knowledge that occurred within each module. To quantify this aspect, the science and practice leaders were asked to display their perception of the degree of TD in percent. Regarding the outputs, the leaders first identified the outputs and afterwards ranked their perceived relevance on a scale from 1 (lowest) to 5 (highest). For analyzing the intangible effects and the TD process as such, the following three blocks of questions were reflected upon.

Collaboration

- How did you perceive the co-leadership of the project?
- Which aspects of the project (e.g., outputs) were particularly relevant from the point of view of the energy region, and the point of view of science?
- What were the most important milestones of the collaboration and why?
- Where did you see problems in the TD process?
- Where do you see improvement potential in the TD process?

Results

- In your view, what were the most important results of the project?

Implementation

- Which results were implemented how well?
- What were problems in the implementation/barriers/hindrances?
- Which additional information of results would you have needed?
- Where is here the improvement potential?

## The case study

### The study area

The energy region Weiz-Gleisdorf (EWG) is located about 20 km east of the city of Graz, has 41,800 inhabitants and a population density of 158 people per km^2^ (BEV 2012). It covers an area of 264 km^2^, with 44 % of the area being used for agricultural purposes and another 42 % as forest area. In 1996, EWG, a federation of 18 municipalities was founded. In 2005, it became EU LEADER region for the period 2007–2013, which provided funding for a management position, who became the co-leader from practice in the TERIM and iEnergy projects. In 2010, the EWG Energy Charta was signed by all mayors setting out a common vision to become CO_2_ neutral by 2050. Within Austria, EWG was declared a “climate and energy model region.” EWG’s focus has been on developing flagship projects in the area of housing and mobility, creating incentives through municipal subsidies and regulations, coordinating educational programs, including social and cultural organizations in the communication process, and implementing diverse promotion activities (Energieregion Weiz-Gleisdorf 2007, 2010). For more information see Hecher et al. (forthcoming).

### Project setting and projects design

**Fig. 2:**
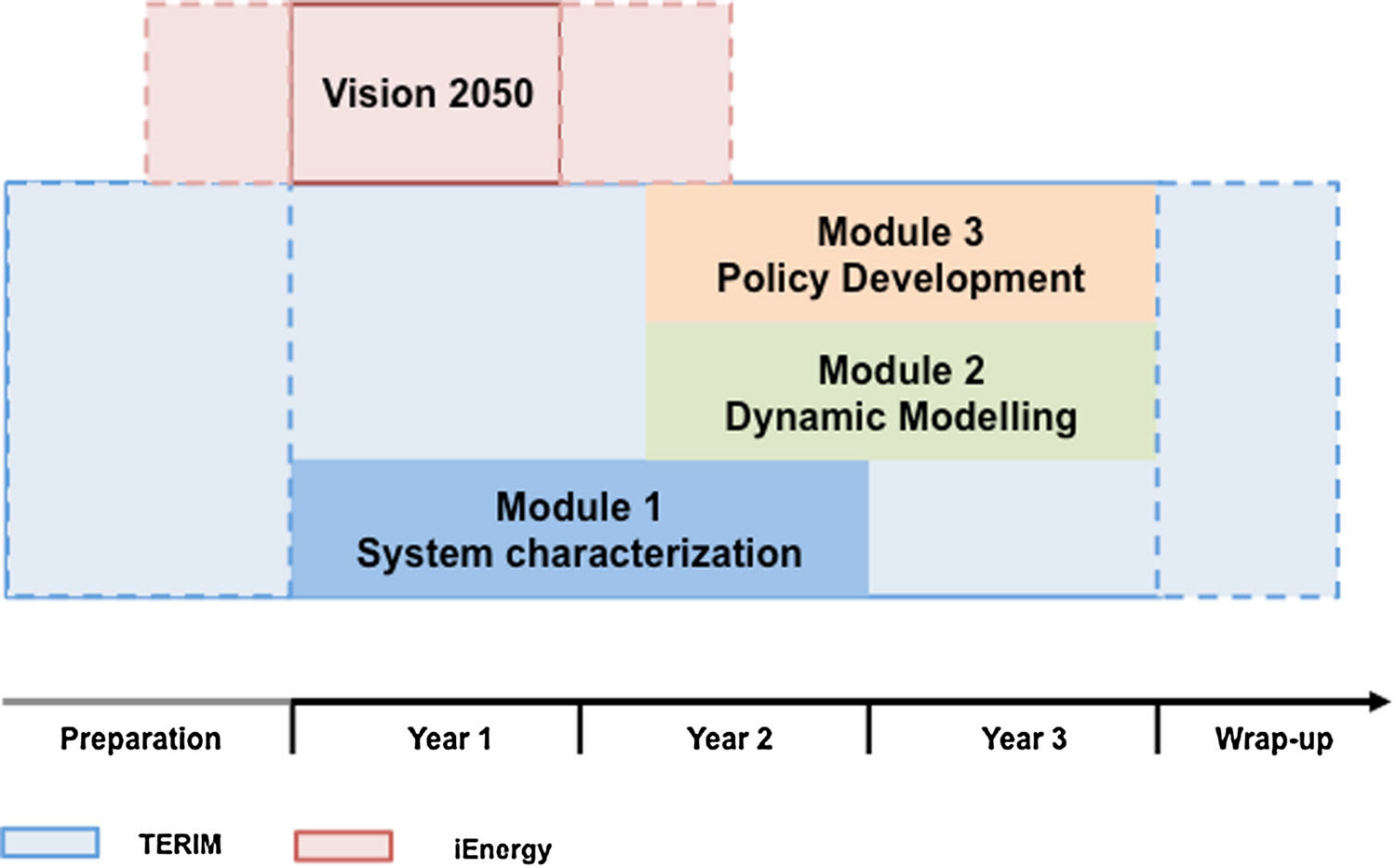
Interaction between the two projects TERIM und iEnergy

The project setting analyzed for this paper consisted of two separate projects—TERIM and iEnergy—which were closely related to each other and which overlapped for almost 2 years of the total project duration (see Fig. 2). Both projects originated from calls of the Austrian Climate and Energy Fond. The first call (ACRP—3rd Call), which provided funding for TERIM, was aimed at scientist as project leaders. The second call (1st call Smart Energy Demo- Fit4Set) was intended to support research consortia led by practice partners in their efforts to develop proposals for the EU based on practice-relevant scientific results.

#### TERIM: transition dynamics in energy regions: an integrated model for sustainable policies

##### Initiation and organizational structure

TERIM was initiated by science (Prof. Claudia R. Binder (CRB)) and co-developed together with the practice leader, Dr. Iris Absenger-Helmli (IAH), manager of EWG.

**Fig. 3:**
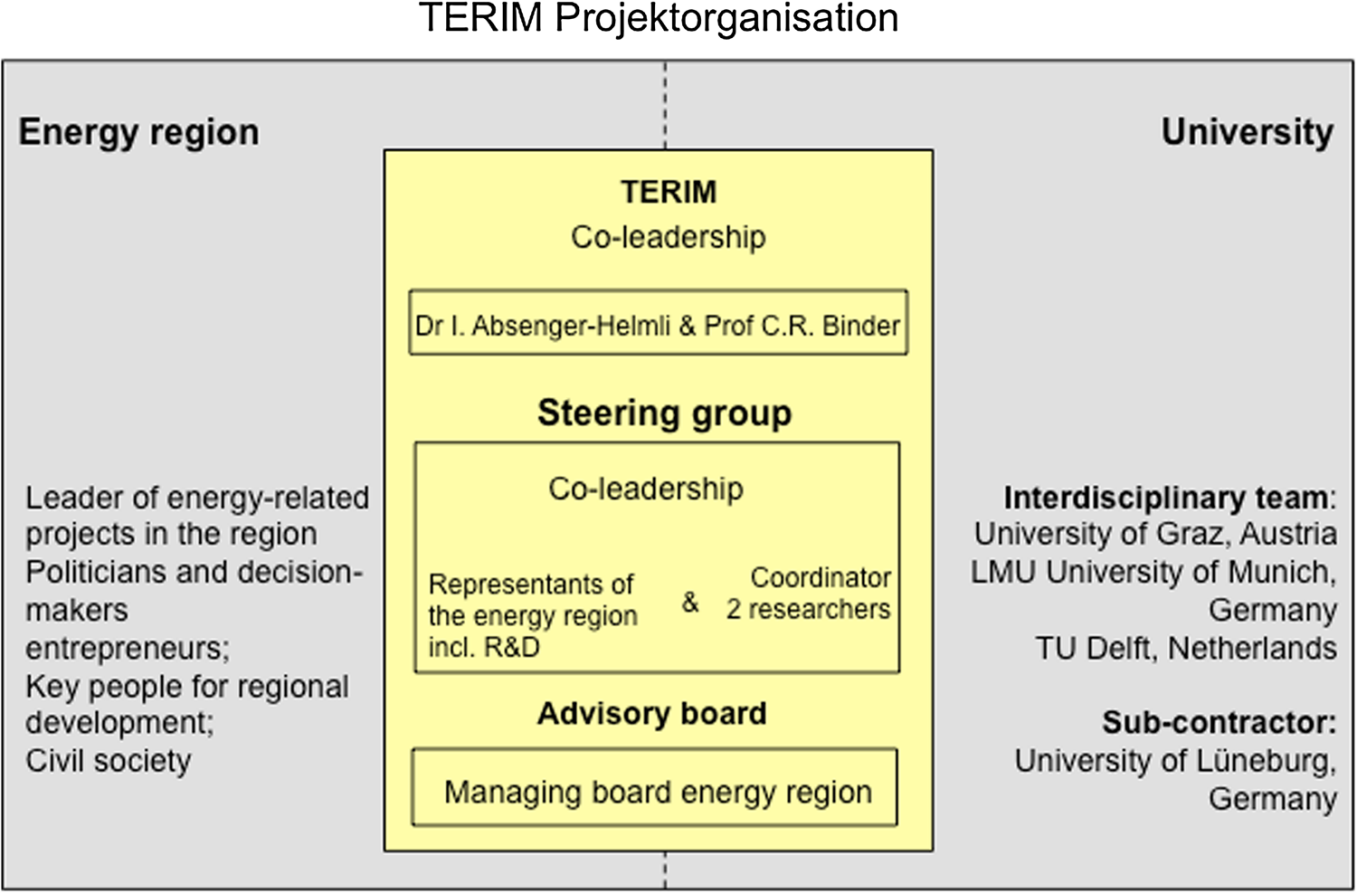
Organizational structure of the project TERIM

Figure 3 outlines the composition of the project team. It shows a double-wing organizational chart representing the co-leadership between science and practice (Scholz and Steiner 2015, this issue). CRB and IAB agreed on a non-formalized co-leadership including an informal agreement on the division of responsibility. CRB was responsible for the quality of the scientific activities, and IAH was responsible for the activities within the region, for the connection to relevant actors, and for assuring that the questions of the EWG would find their way into the project. As such, IAH proposed the composition of the board of management for the project from the practice side, and CRB did the same from the scientific side. Based on the thematic focus of the different work packages, both leaders agreed that additional actors could be called upon to assist the meetings.

During the whole project (incl. the preparatory phase) several formalized meetings took place (see Table 1). The central event in the preparatory phase was the presentation and discussion of the project ideas with the managing board of EWG, to ensure that the project meets the interest of EWG representatives before finishing and submitting the proposal (Table 1). Furthermore, non-formalized meetings and phone calls ensured constant communication during the entire project. The frequency of interaction between the leaders depended on the stage of the project (up to 1 phone call per month).

**Table 1:** Time schedule and overview of project meetings and delivery of publications

Date	Involved people	Topic
Preparatory phase		
18.1.2011	IAB, KB	First contact
24.2.2011	IAB, CRB, KB	Project goals and organization
11.4.2011	EWG board	Presentation of the project
Research phase		
2.5. 2011	IAB, CRB, KB	Preparation of kick-off
4.5. 2011		Kick-off
5.5.2011	Steering group meeting	Project planning
7.7.2011	Steering group meeting	Validation of milestones
12.4.2012	Meeting with partners	Expert interviews, energy cadastre
31.5.2012	Steering group meeting	Intermediate presentation First ideas household survey
30.6.2012	Steering group meeting	Setting the household survey
2.5. 2013	Meeting with partners	Energy cadaster
24.9.2013	IAH, CRB, UV	Planning final work-shop
25.9.2013	Final work-shop	Presentation of results Development of policies
25.9.2013	Steering group meeting	

*IAH* Iris Absenger-Helmli, *CRB* Claudia R. Binder, *KB* Katja Bedenik (Ph.D. student), *UV* Ulli Vilsmaier (Post-doc and later project partner from Leuphana University)

##### Motivation and goals

Energy Regions are regional initiatives in Germany and Austria that strive for energy self-sufficiency through the use of regional energy sources and the development of decentralized energy infrastructure. They are seen as important players in the transition towards a renewables-based energy system in their respective countries. Energy Regions have been reported upon and several manuals have been developed; they have only recently attracted attention from scientists.

The overall goal of the TERIM project was to understand and model the transition dynamics of two Austrian Energy Regions and to derive policy recommendations for establishing new, supporting current, and maintaining successful transitions of Energy Regions. The research aimed to make a major contribution to understanding the dynamics of the transition process, and the role of stakeholder interaction and policy affecting these dynamics. To this end, two cases were analyzed: the Energy Region *öko*Energieland and Energy Region Weiz-Gleisdorf (EWG).

##### Project organization and methods

**Fig. 4:**
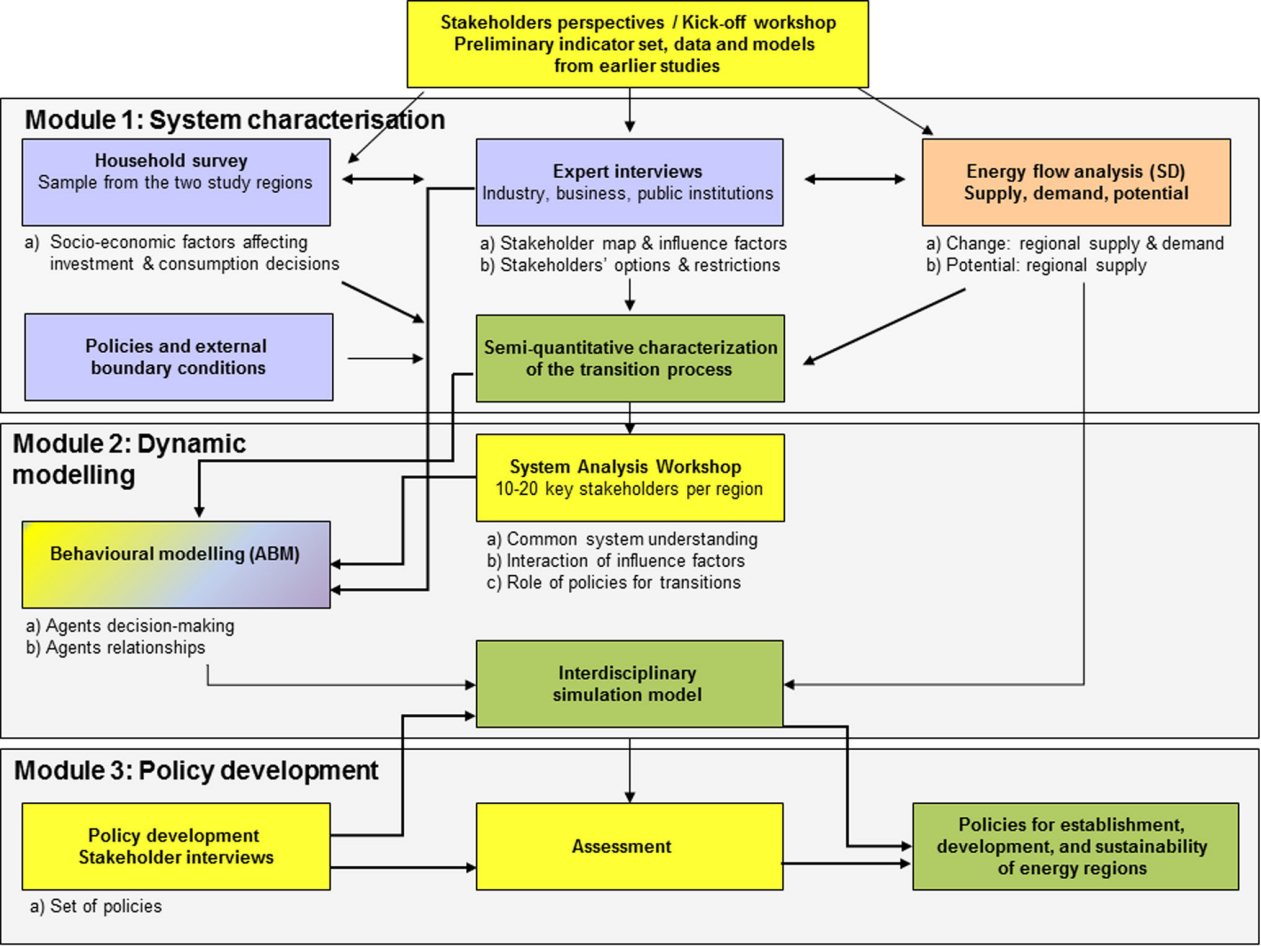
Project structure, modules, and methods of the project TERIM (*yellow* transdisciplinary elements; *pink* social science methods; *orange* natural science methods; *green* results)

The project was organized into three modules: (1) system characterization; (2) dynamic modeling; and (3) policy development. Each of these modules had a different degree of TD and included a specific set of methods (Fig. 4).

*Methods* For characterizing the energy transitions of EWG, we based ourselves on regional study reports (EEE 2011; Energieregion Weiz-Gleisdorf 2007) and gathered data from statistical institutes (Statistics Austria—STATcube Database, BEV 2012). We also applied methods from social as well as natural sciences. The characterization included the (1) development of the physical resource base of the region (e.g., agricultural and forest area, areas covered with photovoltaic cells); (2) development of energy demand; (3) industrial development in the region (e.g., new energy-related firms); (4) socio-economic factors affecting households’ investment and consumption decisions; (5) policies and external boundary conditions; and (6) network of actors and institution development.

We conducted an energy flow analysis (EFA) for the reference years 1990, 2000, and 2010. Thereby, the regionally produced energy (i.e., renewable energy carriers) was compared with the energy consumed in the region resulting in the degree of energy self-sufficiency for each reference year. Detailed data about the region’s energy resources, infrastructure, and demand, including the size, technical standard, and development of the regional building stock, were collected. These were used in a quasi-stationary and dynamic EFA to simulate energy demand and supply given specific policy scenarios (Binder et al. 2014a).

To analyze the transition process in the region we coupled the EFA with an agent and institutional analysis. We defined milestones of the transition process and categorized them into visionary, institutional, physical, and external institutional milestones. Visionary milestones were defined as consolidation of guiding ideas; institutional milestones as permanent and binding agreements of varying degrees; physical milestones, as infrastructural measures in the energy sector; and external institutional milestones as external events affecting the energy regions’ development (Hecher et al. forthcoming). This analysis was validated with the steering board of the TERIM project.

To analyze the social, economic, and political factors affecting the investment and consumption decisions of households related to their energy consumption, we carried out a detailed analysis of the factors affecting householders’ decisions regarding their choice on energy efficiency when renovating or constructing new houses. For this purpose, we combined expert interviews with an in-depth household survey. Because 87 % of total household energy demand accounts for heating and hot water supply, we focused on decision-making related to the energy performance of dwellings, which is affected by policies, personal factors, as well as experts in the building sector. We limited the survey to owners of single-family houses, which cover 80 % of all buildings in EWG (Binder et al. 2014a). Finally, policy scenarios were developed with the partners in the region, and subsequently tested with the simulation model (Binder et al. 2014a; Knoeri et al. 2014).

#### iEnergy: citizens supported by a stakeholder process implement intelligence to upgrade their smart urban region

##### Initiation and organization structure

**Fig. 5:**
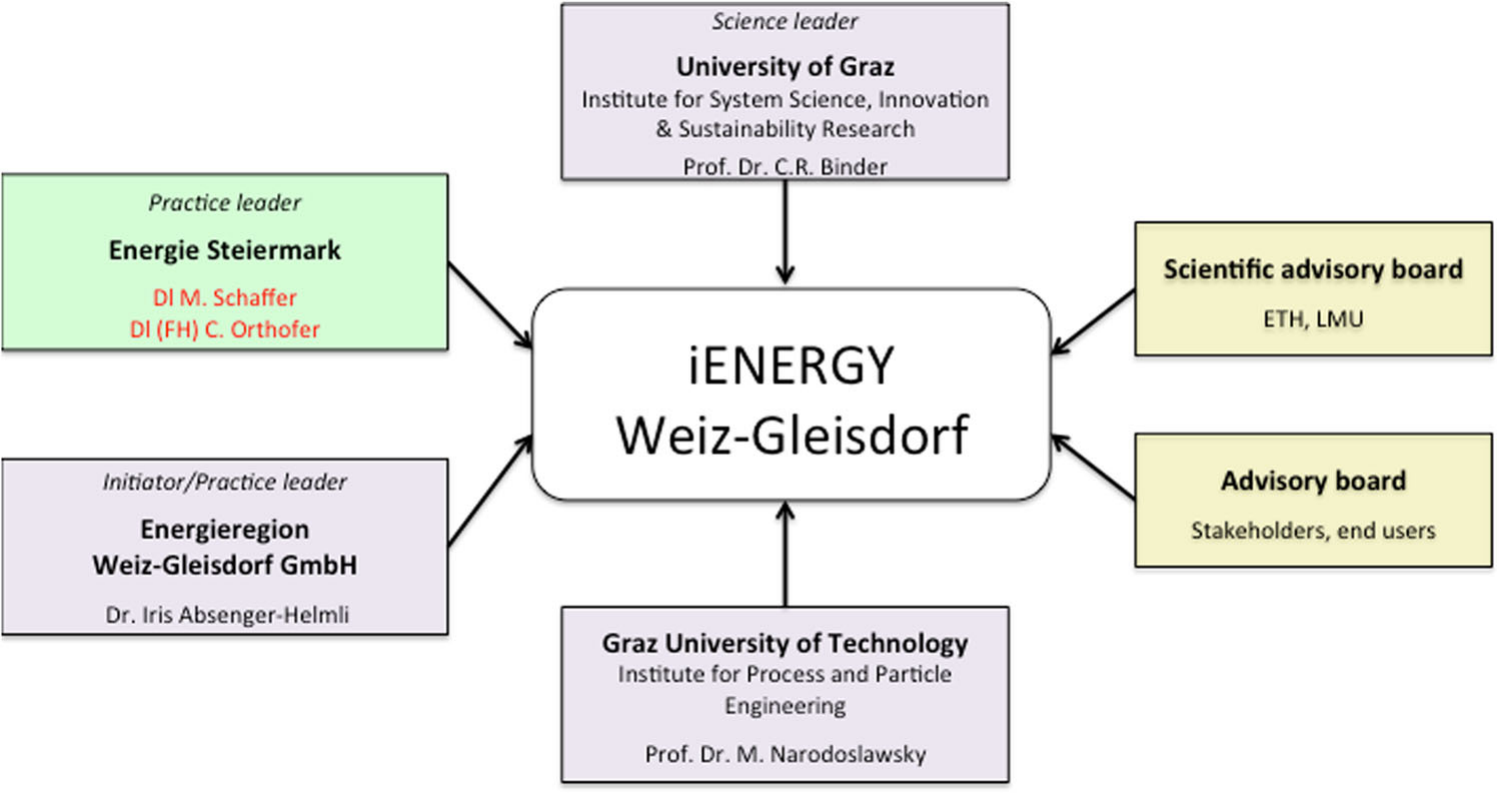
Project consortium of the project iEnergy

iEnergy was initiated by the practice leader IAH at EWG and coordinated by M. Schaffer from Energie Steiermark (regional energy supply company). CRB was the scientific co-leader heading the scenario- and vision-building process and IAB was the co-leader in the region. The Technical University of Graz was an additional scientific partner responsible for the technical part, i.e., bringing knowledge on technical development and energy efficiency to the project. Furthermore, representatives of the steering group of TERIM participated in some parts of the iEnergy project (Fig. 5).

##### Motivation and goals

EWG currently faces the challenges of a growing urban region, such as increasing energy demand, high costs for infrastructure development, and urban sprawl. To ensure the sustainable development of the region, a vision was needed for the further development of the region until 2050. Based on the vision, a roadmap and action plans were developed addressing the areas of communication, information, energy, buildings, mobility, and the interface between people and technology. In this paper, we only consider the transdisciplinary scenario and vision development of the project, which was co-headed by IAB and CRB.

##### Project organization and methods

The transdisciplinary process of developing scenarios for EWG and defining a vision was organized in three steps (Table 2). During the preparatory phase, a transdisciplinary consortium (TD consortium) was established, the project organization was defined, and the project proposal was written (see Scholz and Steiner 2015, this issue). The second phase involved the development of scenarios based on a formative scenario analysis (Scholz and Tietje 2002; Binder et al. 2014a, b). Here, we first developed boundary scenarios, defined as possible future contexts in which the region could be embedded. They were derived from a combination of external impact factors and opened the scenario tunnel by means of their possible minimal and maximal developments (Zah et al. 2010). We then developed system scenarios that captured the effects of a boundary scenario on the region itself (Zah et al. 2010).

**Table 2:** Steps of transdisciplinary (TD) scenario development process, definition of the vision, and stakeholder involvement

Project steps	Involved people			
	TD consortium	Individual experts	Expert group	Population
Preparatory phase (project organization and project proposal)	×			
Formative scenario analysis				
(a) Characterization of the system and selection of influence factors	×	×		
(b) System analysis	×			
(c) Future state of influence factors and consistency analysis	×	×	×	
(d) Development of boundary and system scenarios	×	×	×	
Scenario assessment—definition of the vision	×			×

In Phase 3, the TD consortium selected scenarios and the local population was involved in the assessment of the scenarios leading to the vision (Table 2). A poster was designed for each of the five selected system scenarios, including the current state and the state of EWG in 2050. These posters included five key categories linked to the earlier development plan of the region: (1) resource use, (2) mobility share, (3) energy consumption, (4) green jobs, (5) landscape, and (6) buildings. A timeline illustrated the way to reach each scenario. Challenges and contributions to the goals were clearly depicted in bullet points, to ease the understanding of the scenarios (Schaffer et al. 2012; Binder et al. 2014c).

The scenarios were assessed through the so-called ‘scenario parcours,’ public consultation events that were organized in the two main cities Weiz and Gleisdorf (Scholz and Tietje 2002). Hundred inhabitants participated and were asked to evaluate the scenarios using a green dot for the desired scenario and a red one for the least desirable one. In total, 78.4 % of the participants were male and 21.6 % female while the average age of the participants was 48 years (SD 14). Most participants had a secondary level education and 37.5 % participants had a University degree.

**Fig. 6:**
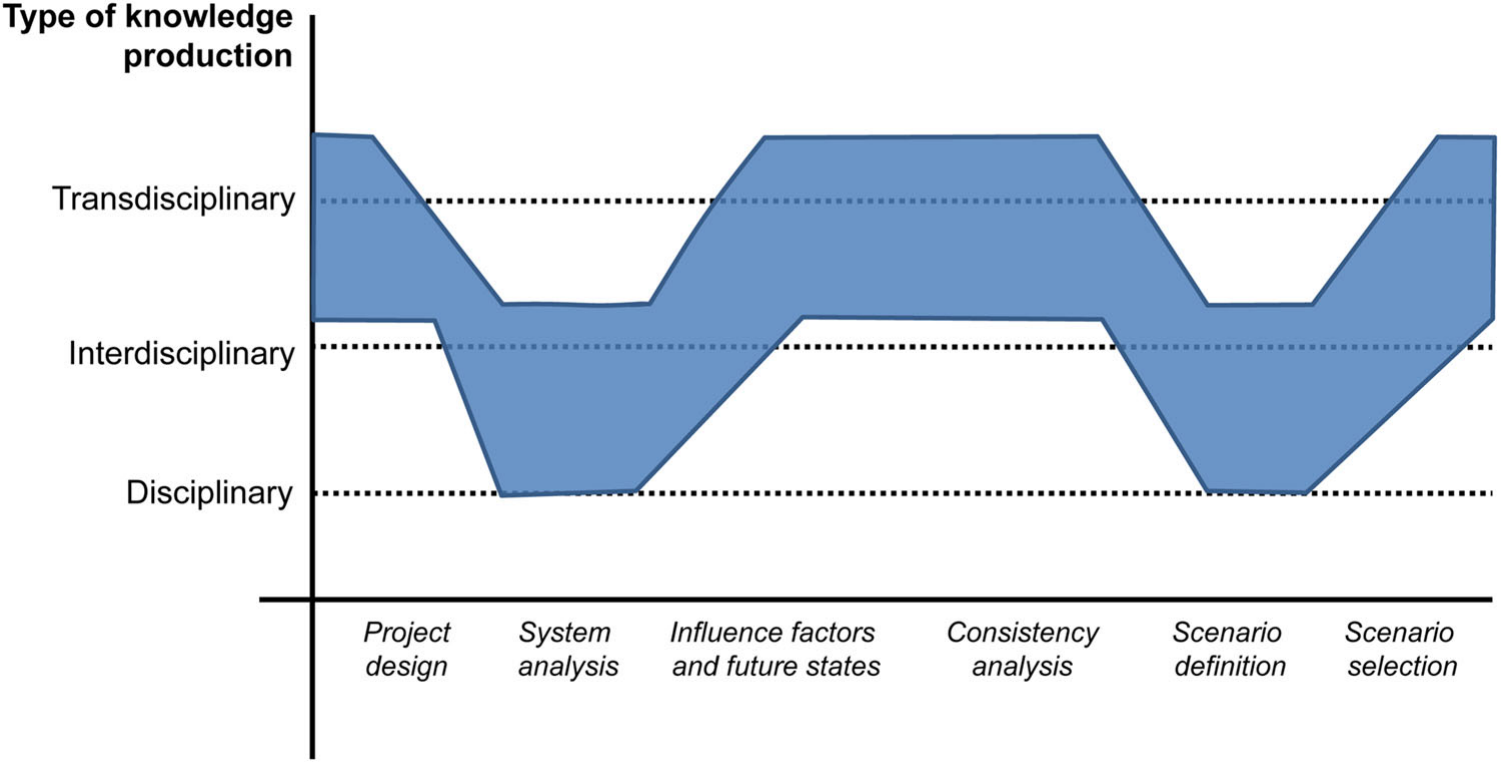
Disciplinary, interdisciplinary, and transdisciplinary steps within the scenario-building process (from Binder 2014)

The different steps of the scenario process relate to disciplinary, interdisciplinary, and transdisciplinary aspects, as shown in Fig. 6 (Wiesmann et al. 2011; Binder 2014). The information gained in the disciplinary and interdisciplinary steps fed into transdisciplinary work-shops. In addition, it would be possible to attribute the intensity of participation in the scenario and vision-building process to the ladder of citizen participation (Arnstein 1969; Stauffacher et al. 2008).

## Results

We structured the results section as follows: First, we provide an insight into the product-related or tangible outputs, impacts, and outcomes generated in each project and project phase. Second, we present the process-related, intangible effects.

### Product-related effects

Table 3 captures the tangible outputs, impacts, and outcomes of the two TD projects.

**Table 3:** Product-related effects of the TERIM and iEnergy project, including a rating of the relevance of the different modules from the perspective of science and practice leaders

Module	TD^a^	Outputs (tangible)	Relevance^b^		Impacts (tangible)	Outcomes (tangible)
TERIM	100 %		Science	Practice		
Kick-of WS						
System characterization	20 %	Institutional analysis—milestones	5	5	System knowledge	
		Validation WS	5	4	Technical	Energy Atlas
		Energy flow analysis (energy infrastructure, energy demand, regional energy sources)	5	4	Physical	Regional planning
		Survey—behavioral analysis	5	5	Social	Comm. strategies
		Survey—perception region	3	4		
Dynamic modeling	30 %	Dynamic building model	5	3	Transformation knowledge	Ex-ante assessment of policies
		Dynamic behavioral model	5	4		
		Dynamic integrative model	5	3		
Policy	80 %	Energy supply	4	5	Transformation knowledge	Calculation on potentials
		Energy demand	4	5		
		Institutional development	3	3		
General						Research projects: EnerTransRuhr, INOLA, RESHAPE, TraNe
iEnergy						
Scenarios	50 %	Scenario work-shop	5	5	System knowledge	Boundary and systems scenarios made visible and tangible for the population
		Scenario report	3	4	Goal knowledge	
		Scenario posters	3	5		
		Scenario parcour & survey	3	5	Transformation knowledge	
Vision	90 %	Definition of vision	4	5		Implementation Almenland
General						iEnergy 2.0^d^

^a^TD: TD component

^b^1: No relevance/5: high relevance

^c^EnerTransRuhr: development of an integrative and transformative research design in the case of the energy transition of the ruhr area and North Rhine-Westphalia. INOLA: Innovations for a sustainable land and energy management at regional level. RESHAPE: Reshaping Institutions and processes in the transition towards renewable energy. TraNe: transformation towards a sustainable energy system: analysis and transdisciplinary modeling of governance processes at regional level

^d^iEnergy 2.0: iEnergy Weiz-Gleisdorf 2.0—the power of a vision

#### Outputs and impacts

The outputs relate to the steps within the different modules and include additional products such as papers and project reports not shown in Table 3. In TERIM, two interim and one final report were produced; 6 project work-shops (linked partly to steering board meetings) were carried out in EWG (see also Table 1); 10 presentations at international conferences were given until the end of 2014; and 4 papers and extended abstracts have been published, three publications are under review and three in preparation. In iEnergy the main output has been the final report. Data and scenarios generated in iEnergy have also been presented within TERIM-related presentations. Regarding the tangible impacts, the module system characterization (TERIM) and part of the scenario analysis (iEnergy) generated *system knowledge*. One result was an in-depth energy flow analysis based on the statistical data from Austria comparing the years 1990, 2000, and 2010. This fed into the energy cadaster developed at EWG. As IAH puts it “…without these results we would not have been able to calculate our energy cadaster and that was fantastic. I have to say if you have something concrete at hand than you really have leverage.” Second, we found that currently, EWG is not able to meet its energy demand from regional sources (the degree of energy self-sufficiency (excl. mobility) with renewables in 2010 was 26 %). In the future, EWG could potentially cover its energy demand if the entire available area (i.e., forest, agricultural, roof, and façade areas) was to be used for generating energy (Binder et al. 2014a). For IAH this was an important insight “A key insight for EWG was—energy self-sufficiency is for us currently impossible—we do not have sufficient resources for producing the energy demanded.”

Third, we found that the transition process is characterized by visionary, institutional, physical (e.g., new infrastructure, connections to heating grids), and external institutional milestones. The visionary and institutional milestones precede the physical ones. A time delay between the vision, the establishment of an institutional governance body, and its impact on the energy system was observed (Binder et al. 2014a, b; Hecher et al. forthcoming). This result did not have a direct impact on EWG itself but is an important information for other regions aiming at transforming towards “energy regions.”

Fourth, our survey of homeowners who had either renovated or built a new house in the last 5 years, revealed that in most cases (66 %) homeowners chose the low-energy house standard B (≤50 kWh/m^2^a) for their building or renovation projects, followed by the conventional standard C (≤100 kWh/m^2^a) (21 %). Only 13 % realized ultra-low-energy, passive or plus-energy houses with higher energy efficiency standard (A (≤25 kWh/m^2^a), A+ (≤15 kWh/m^2^a), A++ (≤10 kWh/m^2^a)). These results have not yet been discussed with our partners from practice.

Regarding *goal knowledge*, iEnergy generated, with a scientific sound procedure, five scenarios that considered different aspects of the region’s development including resource use, mobility share, energy consumption, green jobs, landscape, and buildings. The inclusion of the population was an important step to shape the future process within the region. The vision selected by the local governance body and citizens, “the region flourishes,” has served as a guideline for developing the regional development plan 2014–2020 of the new LEADER-local action group “Almenland & Energieregion Weiz-Gleisdorf.” The vision shows a possible, idealized future and therefore provides the region and its decision makers with the opportunity to examine crucial factors, which have to be addressed in order to realize this transition (see also Trutnevyte et al. 2011). As IAH puts it: “What was relevant? … first of all to understand: what is a vision? What is a scenario? … These are relevant topics for mayors.”

Regarding *transformation knowledge*, we found first that for the regional energy system to be transformed, a reduction in energy demand is essential. Here, changes in the building stock play a central role, i.e., renovation and retrofitting to meet higher energy standards. Our simulation results suggest that there is a trade-off between increasing the renovation rate and higher energy standards (Binder et al. 2014a; Knoeri et al. 2014). We found that the final demand of energy for heating per year is lower if legislation is tightened (as planned in EWG) than if the envelope renovation rate is doubled. However, if we consider cumulative energy savings, doubling the renovation rate would save three times more energy (about 3 TWh compared to the business as usual scenario) until 2050 than a strengthened legislation for envelope renovations would do (about 1 TWh; Binder et al. forthcoming). When we presented these results to our practice partners in order to develop different policy measures in our final work-shop, policy-makers started discussion on how to increase the renovation rate. According to IAH the results showed “what is possible and what is not, and in which areas to develop policies for improving the situation.”

#### Outcomes and leverage

Both projects were the basis for further projects and activities within the EWG and the science partners. In EWG these included four major projects:

*Energy cadaster* Both TERIM and iEnergy underlined the importance of a region-wide database in relation to the actual status of the regional use of energy as a basis for strategic regional energy policies towards higher energy efficiency. The energy cadaster, a sub-project first initiated during the project “Eine Region fährt ab” and now further pursued within the framework of the climate and energy model region project “Start up Energieregion Weiz-Gleisdorf,” addresses this issue by collecting, processing, and providing the therefore needed information regarding space heating, electric appliances and systems as well as private, public, and operational mobility via an online database. The cadaster is based on the data generated from the EFA.

*iEnergy 2.0* “iEnergy Weiz-Gleisdorf 2.0—the power of a vision!” is a follow-up project of “iEnergy 1.0” initiated by the EWG and six other consortium members. It aims to further the EWG’s previously defined vision 2050 by implementing innovative demonstration projects in relation to “Smart Cities” or “Smart Urban Regions,” the use of renewable energy sources, the increase of energy efficient solutions and the realization of regional energetic autonomy under consideration of relevant stakeholders.

*Fusion with the Almenland* The knowledge and experience gained throughout TERIM and iEnergy had an influence on the EWG’s future strategies in general as well as on the merging process with the “Regionale Gemeinschaftsinitiative Almenland Teichalm-Sommeralm”(short Almenland) to form a new local action group within the framework of the European funding program “LEADER.” First interactions with the Almenland started during the LEADER-period 2007–2013, when both regions intensively addressed the topics “renewable energies” and “energy efficiency” on their own as well as via joint projects. During the fusion process both regions strongly agreed to jointly pursue the vision 2050 throughout the upcoming LEADER-period 2014–2020 by developing and implementing participative and interdisciplinary projects and measures. In this regard, the new region benefits from the findings of TERIM and iEnergy in terms of a deep understanding of critical determinants for a successful transition towards a renewable energy system.

“*Start up Energieregion Weiz*-*Gleisdorf*” is a project which is carried out within the framework of the EWG being a climate and energy model region. It aims at taking another step towards realizing the EWG’s vision 2050 by developing an implementation concept and realizing it through pilot projects along crucial development axis. This project also creates the needed conditions to critically review and, if necessary, adapt the vision in order to keep it alive.

Although these long-term developments are ongoing, their initiation is clearly visible and can be related to the TD projects, thereby representing tangible outcomes.

For scientists, the project provided the basis for designing future research projects, as described below.

At the University of Graz, TERIM led to a follow-up project funded by the ACRP on “Reshaping Institutions and Processes in the Transition towards Renewable Energy” (RESHAPE), 2013–2015. It also fed into a work package on energy transition as part of “Transition towards Smart Living Environments,” a project funded by the Province of Styria. In fall 2014, at the University of Graz started a FWF-funded doctoral program on Climate Change Strategies, including one PhD-position on an energy transition-related research topic.

At the Ludwig-Maximilians University in Germany, the project was the basis for two main research projects: (1) Transformation towards a sustainable energy system: analysis and transdisciplinary modeling of governance processes at regional level (TraNe), financed by the Bayrischer Forschungsverbund and (2) Innovations for a sustainable land and energy management on a regional scale (INOLA) is a five-year project financed by BMBF. The latter benefits directly from the experiences made in iEnergy and TERIM because it includes a co-leadership arrangement. Importantly, both co-leaders—science and practice—are funded by the BMBF.

### Process-related (intangible) effects

#### Outputs: experiences during the co-leadership

Both leaders viewed the experiences gained during the TD project in a positive light. Similarly, the co-leadership arrangement was perceived to be on a level playing field. The roles were clearly defined and taken up by both leaders. The practice leader effectively used her knowledge of the region, her experience of working with the mayors of EWG and carrying out projects in the region, and her ability to anticipate possible problems in the region. The science leader contributed her scientific expertise, methodological competence as well as her experience of working in inter- and transdisciplinary projects. In the interview, the practice leader stated it as follows “the (co-leadership) was perfect for me! This is the way I envision things to work. Moreover, I think that being from the region means that I know how things work here. And you [*referring to the science leader*] have your ties in the scientific community. … and I think that we were moving in the same direction. Out of these two leaderships a thick robe emerged—this is the way I would describe it.” From the science leader point of view, the project could not have been developed in the depth if it would not have been for the co-leadership and support in the region.

Each of the leaders stayed within her role during the entire project and they actively communicated if problems emerged. As IAH puts it “and what I think worked well was that we were always in communication—this is going o.k. or where do we need to adjust the project. I think that worked very well.” Evidently, a relationship of mutual respect, trust, and sharing of power developed and the ties between the two co-leaders became closer. As CRB describes it: “… it was great that you said: … Oh, Ms. Binder, here we have to be careful or ‘we need to communicate more’—things that a scientist cannot know from the development of the region.”

#### Cognitive impacts from the experiences

From the practice leader’s perspective, the experiences gained during the project were seen as an added bonus. The knowledge gained of how to work in inter- and transdisciplinary teams has served as the basis for future projects. IAH stated “the other regions are behind us—we are at a different level … we know how to collaborate with universities and how to foresee and analyze problems in transdisciplinary cooperation.” And “we have a better understanding of the system; we understand the interactions between problem areas and system variables” (ibid.). She also stated that the mayors on the managing board agreed with her. From the science leader’s perspective, the experience of successful collaboration reinforced her opinion that transdisciplinary projects, which explicitly incorporate a co-leadership arrangement, do provide a win–win situation for both practice and science leaders. A key insight thus was that it is essential to acknowledge and respect the abilities and the role of the practice co-leader (see also Scholz and Steiner 2015, this issue).

#### Outcome: decision-making capacity

Both project leaders’ capacity to make decisions and to solve problems within a TD project increased significantly. During the iEnergy project a communication problem emerged at the point where the results from the scenario analysis should be made tangible to the population in form of posters, as a first step for the vision development. The communication problem was further exacerbated by time pressure, and misunderstandings emerged. IAH stated “my advertising specialist said: we are in a communication catastrophe. I found the word really accurate. We had to agree on a text that the person in the street would understand and which still would be scientific enough. … I remember your co-worker wanted always to include one sentence more….” CRB added: “we needed a large amount of time for the first part, then part of the team, including myself, moved to Munich [and could not travel often to Graz] which caused further problems … all of the sudden there was this incredible time pressure… I think there was the submission of the follow-up project….” An open conflict resolution procedure, including several meetings and phone calls, resolved the issue, to the satisfaction of all parties involved.

The development of networks also added to the leaders’ decision-making capacity. Both leaders enlarged their networks, and existing collaborations became more intensive. IAH stated that “well I think that TERIM and iEnergy intensified our networks also through providing an understanding of the complexity of the topics and relationships. And you also became aware of whom you could rely on if you wanted to get something done….” Furthermore, the process raised awareness of the complexity of the energy issues among the mayors on EWG’s board of management and the population participating in the visioning process. The science leader built new ties to the scientific partners and an interdisciplinary learning process where both exchanged and learned from their methodologies took place.

## Discussion

This paper captured the self-reflections of two co-leaders, one practitioner and one scientist, on two transdisciplinary projects. In the following, we first discuss the product and process-related effects. Second, we elaborate on the process itself and identify four key elements for a fruitful TD project. Finally, we reflect on the utility of the framework used and present ideas for further research.

### Product- and process-related effects

We presented the different product- and process-related effects encountered during the projects. In the following, we depart from the *tangible impacts*, namely system, goal, and transformation knowledge, and relate these impacts to TD and outreach in practice and science.

The explicit *system knowledge* gathered in both projects, e.g., the data for the energy flow analysis, was of importance for both the practice and the science leaders. For the practice leader, the outreach of the system knowledge data became evident as the follow-up project, the energy cadaster, started. For the science leader, the data were essential for the models developed during the project. Moreover, of key importance for the science leader were not only the results elaborated by the scientists themselves but the in-depth understanding of the processes within the region provided by the practice co-leader and the practice partners in the managing board. This implicit system knowledge was shared during steering committee meetings and through personal conversations. It was crucial for the design of the project, and for understanding the link between institutional development and the technical energy system, and therefore, for understanding transitions of energy regions (Binder et al. 2014a, b; Hecher et al. forthcoming).

The *goal knowledge* developed through the scenario- and vision-building process in iEnergy had short-term effects on several regional projects as are iEnergy 2; “Start up EWG”; and “Fusion with the Almenland”. The practice co-leader perceived it as the highlight of the collaboration, whereas the work-shops carried out to understand the relation between the system elements, to analyze the consistency of future states of the impact factors, and to decide upon which scenario should become a vision, were viewed best. These steps had also the highest degree of TD (Table 3). Furthermore, both leaders stated that the process of scenario building contributed to trust and network building and to mutual learning. However, the scenarios as such did not deliver sufficient information for organizing the transitions process; they were not directly implementable. IAH said: “the more you get into the issue, the more you realize: the vision is nice but can we pay for it? How can we implement it? Is there really a desire for implementation?”

From the point of view of the science co-leader, the scenario- and vision-building process was important for getting an insight into the perspectives of the practice partners. The results obtained were fed into the last research step, the simulation and assessment of policies.

The *transformation knowledge* increased the decision-making capacity of the practice leader and partners. First, practice partners were able to distinguish what would be possible in the near future and what might happen at the long term. Second, the results provided the basis for long-term planning as the potential effects of policies became visible. Here, simulation results played the same role as a backward planning process (Trutnevyte et al. 2012).

### Lessons learned from the TD process

Our self-reflection also provides some useful inputs regarding factors that either help or hinder a fruitful TD project. We highlight critical aspects found during the co-leadership of the two projects and partly reflect on the factors found by Scholz and Steiner (2015, this issue).

#### Timeliness of research

When reflecting upon the relevance of the TD project for practice, an important issue was raised by the practice co-leader. For her, it was not only important that the project was developed in co-leadership but that it also was developed at the right time. She stated that she carried out several projects in the past and that a proper flow was very important. This flow occurred whenever a project was timely and involved close collaboration. Considering further collaborations IAH stated that “well… currently it would be a little bit too early… well, we still need about three quarters of a year… but then scenarios, or an energy cadaster … that would be great.”

That is, for a TD research to be timely, two issues seem to be of relevance. First, if the research interests and the needs from practice partners overlap—at least to some degree—this will yield benefits for science and practice (proposition 3 by Scholz and Steiner 2015, this issue; Pohl et al. 2010). This implies that the choice of the system and research topic have to be scientifically innovative and at the same time, be sufficiently ill-defined so that actors from practice want to become involved (Scholz and Steiner 2015, this issue, Table 1; 1.1). Second, the science and practice co-leaders should have sufficient institutional support. This means that scientists as well as practitioners are well embedded into their respective organizational structures and supported in their aims through them (Scholz and Steiner 2015, this issue, Table 1; C1.7). One way for facilitating this is to set up a steering board in practice and science so that the respective organization can follow, support, and comment on the project, increasing its embeddedness in the region and within science. If the project, however, is not timely, it might be hampered as described by Antrop and Rogge (2006, p. 389): “also the formulation of visions for landscape management was dropped as the active involvement of the local stakeholders was not yet realized at this stage.”

#### Accept that science and practice are different

A second aspect that emerged during the self-reflection was that one has to accept that science and practice are different (Scholz and Steiner 2015, this issue, Table 1; C1.6; 1.2.3). This implies: (1) to take seriously the needs of both science and practice and to try to align them. This means, first, to accept that the relevance of the different research steps is not equal for science and practice leaders (Table 2). Second, it requires the openness to readjusting research questions and methods not only due to the research process but also because of changes in the needs of the practitioners or the political environment. (2) to recognize and accept that not every step in the project has to be performed together and in a transdisciplinary way (Wiesmann et al. 2011; Binder 2014; Stauffacher et al. 2008). That is, purely disciplinary research has to be carried out in between transdisciplinary phases. Moreover, activities within the region have to be pursued without input from scientists. This has to be accepted by both leaders. (3) to be aware that long-term leverage and outreach are extremely relevant, in addition to direct and immediate effects of the TD process itself. This has to be taken into account in the planning of the project (Scholz and Steiner 2015, this issue, Table 1; 2.2.1; 4.1.2).

#### Truly lived co-leadership and trust

Both leaders of the projects agreed that the key to the success of their projects was a truly lived co-leadership 'on a level playing field’. This co-leadership built their mutual trust and strengthened their ties during the projects. This is reflected by a statement of IAH “No, if I cannot stand behind a project, I cannot stand in front of my people and say… this is it. I would feel ashamed, because I am the person standing in front of EWG, you know?… I am the person representing the region.” This aspect is also stressed by (Böhm 2006) who emphasizes a trustful interaction as success factor for similar project settings. The truly lived co-leadership included: (1) to clearly define the roles and competencies from the beginning and for leaders to take ownership of these roles (see proposition 1 and Table 1; 1.2.1 Scholz and Steiner 2015, this issue; Mauser et al. 2013; Wiek 2007; Carew and Wickson 2010). This leads to clearly defined power relationships within the project; (2) to clearly and cooperatively state the research question and set the project goals (Defila and Di Giulio 2006; Scholz and Steiner 2015, this issue, Table 1; 2.1.2); and (3) to understand from a science perspective that it is important for practice leaders to see the benefits of the research for their own region. This implies that some knowledge generated during the project may not lend itself to scientific publications which are relevant to science leaders. Leaders and partners from practice may also gain knowledge that they initially considered to be irrelevant but that will give them a more in-depth view of the processes and interrelations within the region (see above).

#### Communication and language

Proper communication was crucial for the entire TD process, confirming previous research findings (Wickson et al. 2006; Carew and Wickson 2010; Bergmann et al. 2005; Böhm 2006). As described above, and in agreement with other scholars, communication problems are likely to exacerbate whenever there is time pressure, and should thus be planned for (Antrop and Rogge 2006; Defila et al. 2006; Bergmann et al. 2005; Lang et al. 2012). In our case, regular telephone conferences and meetings between the two co-leaders to share the developments in the scientific and practice parts of the projects proved to be essential. As a result, potential problems due to political changes in the region could be anticipated and overcome without endangering the project itself. The fact that both leaders had good communication structures within their respective teams proved extremely valuable when misunderstandings emerged between the teams due to time pressure. Regarding communication within the steering group, content-related work-shops that offered opportunities to present and discuss results and policy options and to elaborate the consistency matrix during the scenario-building process were important for building trust, increasing understanding, and furthering implementation.

This implies that in any TD project (and also interdisciplinary projects), sufficient attention has to be given to language choice and to internal and external communication (Defila et al. 2006; Zscheischler et al. 2014; Wittmayer and Schäpke 2014; Wickson et al. 2006). Clearly scheduled meetings via phone and in person are essential to be aware of upcoming problems and avoid misunderstandings. In larger projects involving several scientific and practice partners it might be even necessary to have people external to the project to support communication and, if necessary, mediation of the process (Böhm 2006; Wiek 2007; Scholz and Steiner 2015, this issue, Table 1; 2.1.6; Hirsch Hadorn et al. 2008b).

### Utility of the framework applied

The framework provided supported the science and practice leaders in their efforts to structure their self-reflection and analysis. Even though the distinction between outputs, impacts, and outcomes was difficult to make, it nevertheless supported their reflections on the short-, medium-, and long-term effects. Furthermore, the distinction between process- and product-related effects supported the leaders’ reflections on personal issues and relations, drawing attention to previously unconsidered issues and factors. However, as already mentioned by Walter et al. (2007) the measurement of the intangible effects proved to be a difficult task. This is in particular true as tangible outputs or impacts (e.g., goal or transformation knowledge) might increase the decision-making capacity of the practice partners, which is an intangible outcome. Walter et al. (2007) solved this problem by using a quantitative statistical procedure in which the different effects were correlated to each other. In qualitative analyses, as in the presented paper, we have inferred on the relation between these effects. There is ample room for research in this respect.

### Further research

The results of the self-reflection presented in this paper open the room for further research: First, we consider that the framework for reflecting TD projects from the view of practice and science co-leaders has still some room for improvement. On the one hand, it should be further developed to capture the views from additional persons involved in the project from science and practice side. Thereby, we hypothesize that the product-related effects are viewed differently by the different actors. This might also affect the way TD projects are structured and lived. On the other hand, tools should be developed to assess and support ongoing projects in shaping their project design in view of increasing impact in practice.

Second, criteria should be developed, to capture the relevance of the different types of knowledge (i.e., system, goal, and transformation knowlegde) for the practice and science co-leaders and partners. As shown above, the relevance of the different types of knowledge was perceived differently and had different impacts on the practice and scientific components of the projects. This analysis could be furthered by differentiating between producing knowledge advancing the scientific debate and producing knowledge advancing practical solutions. The following questions could be tackled: What is the effect for scientific partners of having to produce knowledge that might not lead to publications? and What criteria support assessing the relevance of knowledge?

Third, further research on transdisciplinary research projects should focus on the factors affecting the transformative power of transdisciplinary research. As suggested by our results, the timeliness of the research, with its implications for science and practice, is essential for generating outputs and impacts, which will produce long-term tangible and intangible outcomes (also for practice partners). The questions that emerge are: When does a transdisciplinary project have transformative potential? Which boundary conditions might support or hinder the potential to come true? Is there something like a cost-income-ratio?

## Conclusion

This paper provided results of a framework-based self-reflection process conducted by the science and the practice leaders of two transdisciplinary projects realized in co-leadership from 2011 until 2014. We analyzed the tangible and intangible outputs, impacts, and outcomes which were elaborated during the project.

The project was reflected positively by both the practice and the science leaders. The gains seen in the co-leadership of these TD projects can be highlighted as follows: “From a scientific perspective the in depth understanding of the processes within the region provided by the practice co-leader and the practice partners in the managing board was crucial for project design and development.” Practice leader [in political competition with other actors]: “the other regions are behind us—we are at a different level … we know how to collaborate with universities and how to foresee and analyze problems in transdisciplinary cooperation.”

Four aspects were seen relevant for the success of the transdisciplinary project: First, the timeliness of the research, including (1) the need of the perspective of the practice partners and the scientific community, (2) the willingness of the co-leaders to develop the project together, and (3) the fundamental organizational support, proved essential for the successful start and development of the TD project. This also implies the need for a high overlap between the interests of the practice and the science leaders, which is likely to lead to a win–win situation where the mutual learning processes lead to mutual gains.

Second, accept that science and practice are different, including (1) to take seriously the needs of both science and practice and to try to align them; (2) to recognize and accept that not every step in the project has to be performed together and in a transdisciplinary way; and (3) to be aware that long-term leverage and outreach are extremely relevant, in addition to direct and immediate effects of the TD process itself.

Third, a truly lived co-leadership leads to a trustful cooperation in which problems during the project can be addressed and solved. This includes (1) clearly defined and lived roles and responsibilities, (2) common definition and alignment of the goals, and; (3) understanding the relative relevance of the outputs for practice and science; e.g., including the need of practice leaders to see the benefits of the research for their own region.

Fourth, a good and well-established communication structure between the project leaders and within the teams is important so that agreement on project content and development can be obtained. Creating a common language is essential and time should be taken for doing so. Furthermore, one has to be aware that communication problems are likely to exacerbate whenever there is time pressure, and should thus be planned for. Finally, one should consider that communication requires time and language has to be adapted in order to fulfill both the needs of sciences and of practice.

Furthermore, one has to be aware that the outputs and impact generated can be valued differently by practice and science leaders. While system knowledge is likely to be the basis for the scientific development of the project, the effects for practice might only be noticeable at a medium term. Goal knowledge in the form of scenarios and visions, on contrary, is felt by the practice partner to have an outcome at the short term, but its implementation might be at long term when sufficient transformation knowledge has been generated.

From a methodological point of view, we went beyond the existing literature on self-reflective TD case studies by grounding our reflection process on a conceptual framework. This approach proved to be helpful to achieve a broader, more holistic perspective while reflecting on the TD projects. Since a self-reflection is by definition a project-internal affair lacking an objective, external perspective, the application of a conceptual framework can help to create distance to the project, structure the thinking process and make it comprehensible, and increase the comparability and reliability of the reflection. Future research should further develop criteria mostly for measuring intangible outputs, impacts, and outcomes.
